# Rapid automated superposition of shapes and macromolecular models using spherical harmonics

**DOI:** 10.1107/S1600576716005793

**Published:** 2016-05-16

**Authors:** Petr V. Konarev, Maxim V. Petoukhov, Dmitri I. Svergun

**Affiliations:** a Laboratory of Reflectometry and Small-Angle Scattering, A.V. Shubnikov Institute of Crystallography of Federal Scientific Research Centre ‘Crystallography and Photonics’, Russian Academy of Sciences, Leninsky prospekt 59, Moscow, 119333, Russian Federation; b Hamburg Outstation, European Molecular Biology Laboratory, Notkestrasse 85, Hamburg, 22607, Germany

**Keywords:** small-angle scattering, protein structure, model superposition, scattering amplitudes, *SUPALM*

## Abstract

A rapid algorithm to superimpose macromolecular models in Fourier space is proposed and implemented (*SUPALM*). The method uses a normalized integrated cross-term of the scattering amplitudes as a proximity measure between two three-dimensional objects. The reciprocal-space algorithm allows for direct matching of heterogeneous objects including high- and low-resolution models represented by atomic coordinates, beads or dummy residue chains as well as electron microscopy density maps and inhomogeneous multi-phase models (*e.g.* of protein–nucleic acid complexes). Using spherical harmonics for the computation of the amplitudes, the method is up to an order of magnitude faster than the real-space algorithm implemented in *SUPCOMB* by Kozin & Svergun [*J. Appl. Cryst.* (2001 ▸), **34**, 33–41]. The utility of the new method is demonstrated in a number of test cases and compared with the results of *SUPCOMB*. The spherical harmonics algorithm is best suited for low-resolution shape models, *e.g*. those provided by solution scattering experiments, but also facilitates a rapid cross-validation against structural models obtained by other methods.

## Abstract 2

A method utilizing a spherical harmonics representation of the scattering amplitudes for a rapid superposition of three-dimensional models is developed.

## 1. Introduction   

Small-angle scattering of X-rays and neutrons (SAXS and SANS) is more and more actively employed for structural studies of biomacromolecular solutions, including hybrid modelling in combination with other structural methods (Svergun *et al.*, 2013 ▸). The SAXS/SANS analysis methods provide three-dimensional models of different nature and resolution, and comparisons between such heterogeneous models are often required for cross-validation of structural results obtained by different techniques. The comparisons usually require automated best-matching superposition of three-dimensional structures. Superposition of heterogeneous models, which may not only be different in resolution and representations (atomic models, bead models, dummy residue chains, density maps, surfaces *etc.*) but also contain parts with significantly different density (*e.g.* nucleoprotein complexes), is not a trivial task.

We previously developed a program *SUPCOMB* (Kozin & Svergun, 2001 ▸) for matching high- and low-resolution three-dimensional structures, which uses a normalized spatial discrepancy (NSD) as a proximity measure between the objects. For every point (bead or atom) in the first model, the closest neighbouring point in the second model is found, and the same is done for all points in the second model. The squared proximal distances are added and normalized against the squared average distances between neighbouring points for the two models, yielding the NSD value for the given relative positions of the models. Starting from an inertia-axes alignment, the algorithm minimizes the NSD and finds the best-matching alignment of the structures. However, the CPU time used by *SUPCOMB* is proportional to the product of the number of points (beads or atoms) representing the two objects and for large macromolecular complexes the program becomes computationally expensive.

There are a number of fitting algorithms and programs in electron microscopy (EM) to fit high-resolution models into lower-resolution electron-density maps, such as *SITUS* (Chacón & Wriggers, 2002 ▸), *FRM* (Kovacs *et al.*, 2003 ▸), *FOLDHUNTER* (Jiang *et al.*, 2001 ▸), *COAN* (Volkmann & Hanein, 2003 ▸), *NORMA* (Suhre *et al.*, 2006 ▸) and *ADP_EM* (Garzón *et al.*, 2007 ▸). All these methods use a real-space cross-correlation coefficient as a proximity measure between the models, some of them (*e.g. ADP_EM*) utilizing a spherical harmonics expansion of the density function. Most algorithms employ an exhaustive search of the six-dimensional parameter space (three rotations and three translations) using different mathematical transformations to speed up the process (fast Fourier transforms and/or spherical harmonics), while some of them perform rapid local optimization algorithms making use of the score gradient (*e.g.* steepest ascent). The above methods are similar in performance and the accuracy of the results largely depends on the software implementation.

Here, we propose an algorithm for a fast matching of macromolecular models based on the spherical harmonics representation of the scattering amplitudes in Fourier space. The method uses a normalized integrated cross-term of the scattering amplitudes as a proximity measure between two low-resolution three-dimensional objects. The method is implemented in a program which is significantly faster than *SUPCOMB* and is directly applicable for comparisons of low-resolution shapes and heterogeneous models of different nature (atoms, beads, EM maps *etc.*).

## 2. A normalized correlation coefficient as a proximity measure between three-dimensional objects   

For an arbitrary three-dimensional object with scattering density ρ_A_(**r**) in real space, the scattering amplitude *A*(**s**) can be represented using spherical harmonics as (Stuhrmann, 1970 ▸)
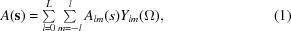
where the momentum transfer *s* = 4π sin (θ/2)/λ with θ the scattering angle and λ the wavelength, Ω is the solid angle in reciprocal space, **s** = (*s*, Ω), and the truncation value *L* defines the resolution. For an object represented by *N* points (atoms, beads or density values) the partial amplitudes *A_lm_*(*s*) are calculated as
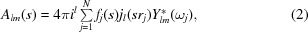
where the sum runs over all elements with coordinates **r**_*j*_ = (*r_j_*, ω_*j*_) = (*r_j_*, θ_*j*_, φ_*j*_) and *f_j_*(*s*) are the corresponding form factors.

Owing to the orthogonal properties of the spherical harmonics, a spherically averaged scattering intensity *I*_A_(*s*) (*e.g.* as measured in a small-angle experiment from an ensemble of randomly oriented particles) is expressed as the sum of individual multipole contributions:
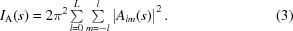
Let us now consider two three-dimensional objects with densities ρ_A_(**r**) and ρ_B_(**r**). The best superposition of these objects should maximize the integral correlation expressed as ∫[ρ_A_(**r**) + ρ_B_(**r**)]^2^ d**r**. Following Parseval’s theorem (Parseval des Chênes, 1799 ▸) this integral is equal to the total intensity in reciprocal space: 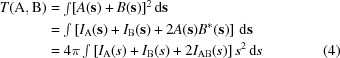
where *I*_A_(*s*) and *I*_B_(*s*) are the averaged scattering intensities of the objects A and B, respectively, and *I*_AB_(*s*) denotes the cross-term. Note that the individual integrated intensities do not depend on the position and only the integral from the cross-term changes when the particles are moved/rotated. Using the spherical harmonics expansion [equations (1) [Disp-formula fd2]–(3)], the three terms are readily calculated in terms of the partial amplitudes and a quantitative measure of the agreement between the two real-space three-dimensional objects can therefore be expressed in reciprocal space as a normalized correlation coefficient (NCC): 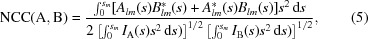
where *A*_*lm*_(*s*) and *B*_*lm*_(*s*) are the scattering amplitudes of the objects A and B, respectively. Here, all integrals are evaluated in a restricted angular range up to *s* = *s_m_* defining the resolution of the objects. Theoretically (for an infinite upper limit of integration), NCC varies between 0 and 1, the latter value corresponding to an ideal overlap of two identical structures. Thus, NCC can serve as a convenient proximity measure and its maximization should allow one to obtain the best overlap of two objects.

## 3. The superposition algorithm for the alignment of two heterogeneous objects   

Numerical minimization of a proximity measure between three-dimensional heterogeneous objects with respect to the positional and rotational parameters is a non-trivial task. The target function may display multiple local minima, where the search algorithms may be trapped. Global minimization algorithms (*e.g*. simulated annealing) can overcome local minima but they are not computationally efficient. A practical approach to solving this problem is the use of a local minimization starting from pre-aligned positions. These can be obtained from the inertia-axes matching procedure as implemented in *SUPCOMB* (Kozin & Svergun, 2001 ▸). Here, the principal axes of inertia are found for both objects as the eigenvectors of the inertia tensor matrix representing linear combinations of the second central moments of distribution around the mass centres. The two objects are set in canonical positions where they are origin centred and rotated so that their principal inertia axes, taken in ascending order of eigenvalues, are aligned along the *X*, *Y* and *Z* axes, respectively. Depending on whether enantiomorph structures are allowed or not, there are four or eight sign combinations of the eigenvalues.

Here, we employ the following algorithm for the superposition of two three-dimensional objects (model A and model B):

(i) Inertia tensors and their eigenvectors are computed for both model A and model B.

(ii) Model B is rotated by **M**_A_ **M**_B_^T^ and shifted by **T**_B_–**T**_A_ to align its principal axes with those of model A (here, **M**_A_ and **M**_B_ are the rotation matrices of model A and model B, respectively, and **T**_A_ and **T**_B_ are the corresponding shifts of the centres of mass from the origin of the coordinate system). The signs of the columns of the rotation matrix **M**_B_ are selected out of four sign combinations (or eight, if enantiomorphs are allowed).

(iii) The scattering amplitudes of the two objects *A*_*lm*_(*s*) and *B*_*lm*_(*s*) are evaluated. Here, one can use *CRYSOL* (Svergun *et al.*, 1995 ▸) for the high-resolution atomic models or *DAM2ALM* [available in the *ATSAS* package (Petoukhov *et al.*, 2012 ▸)] for low-resolution *ab initio* shapes. Options for the direct reading of electron-density maps (EMD files, EMDataBank; http://emdatabank.org/index.html) or multi-phase *ab initio* bead models [obtained by the program *MONSA* (Svergun & Nierhaus, 2000 ▸)] and calculation of their scattering amplitudes are also provided.

(iv) The position and orientation of model B are refined by minimizing the value of 1/NCC using a local minimization by the nonlinear least-squares package *NL2SOL* (Dennis *et al.*, 1981 ▸). The obtained best NCC value is reported as an estimate of proximity measure between the objects.

Steps (i) and (ii) are similar to the ones used in *SUPCOMB*; however, steps (iii) and (iv) are different and offer several advantages. First, the use of the pre-computed amplitudes allows one to easily match heterogeneous objects and models of different nature. Second, the changes in *B*_*lm*_(*s*) upon rotations and displacements of object B are rapidly calculated using the finite rotation elements matrix (Edmonds, 1957 ▸; Svergun, Volkov *et al.*, 1997 ▸). Importantly, the scattering amplitudes are calculated only once and the computational cost of the algorithm does not depend on the complexity of the structures to be superimposed (in contrast to *SUPCOMB*, where the calculation time is proportional to the product of the numbers of elements in the two objects, *N*_A_ *N*_B_).

## 4. Optimal resolution parameters for the NCC calculation   

**Figure 1:**
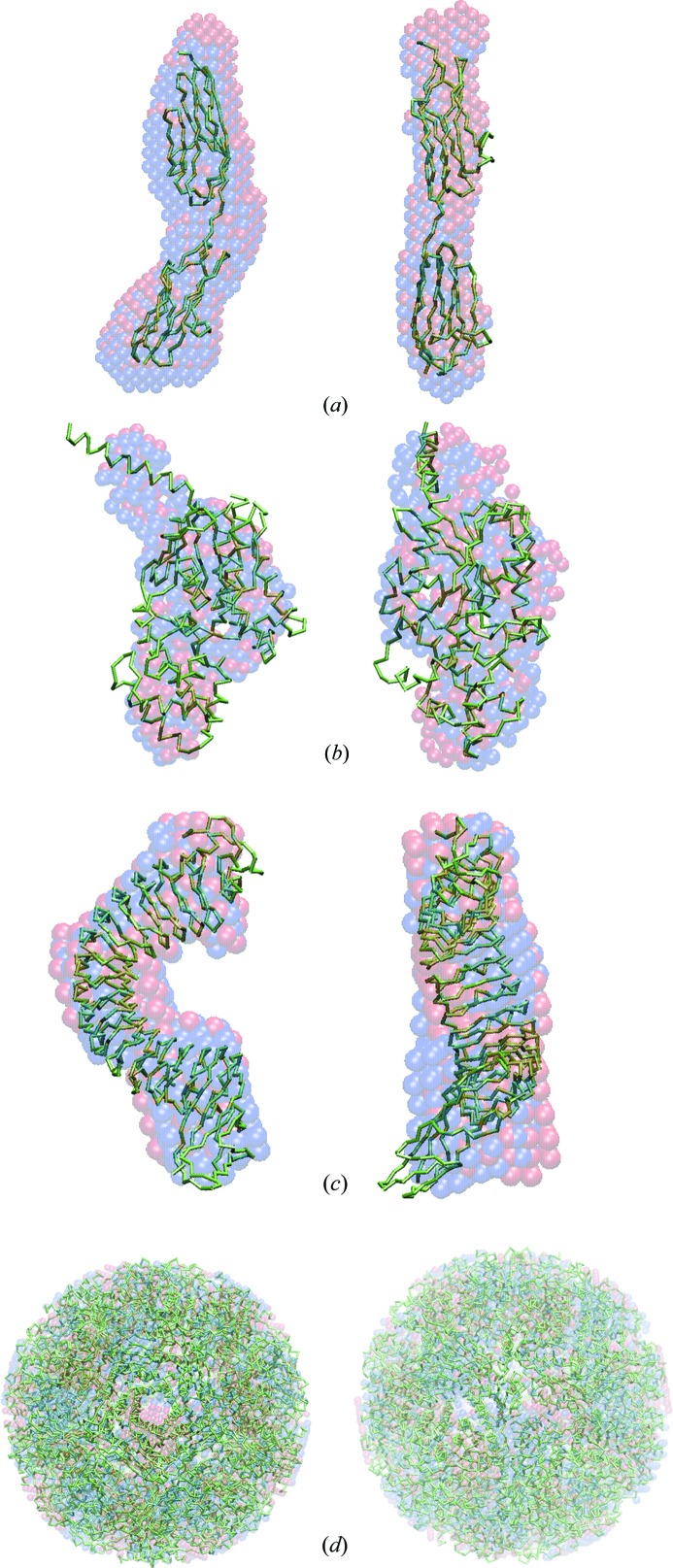
The crystal structures of several proteins (green Cα traces) with superimposed *ab initio* shapes obtained by *SUPALM* (red spheres) and by *SUPCOMB* (blue spheres). (*a*) Z1Z2 protein (PDB code 2a38; Marino *et al.*, 2006 ▸), (*b*) G protein (PDB code 1got; Lambright *et al.*, 1996 ▸), (*c*) internalin (PDB code 1o6v; Schubert *et al.*, 2002 ▸), (*d*) lumazine synthase (PDB code 1hqk; Zhang *et al.*, 2001 ▸). The right view is rotated counterclockwise by 90° around the vertical axis.

The method was implemented in a computer program called *SUPALM*, and its performance was tested in a number of test cases on various high- and low-resolution models of biological macromolecules and compared with *SUPCOMB*. Most of the low-resolution models in Tables 1 ▸ ▸–3 ▸ are experimentally determined *ab initio* protein shapes reconstructed from SAXS patterns measured at the beamlines X33 and P12 (EMBL c/o DESY) using *DAMMIN* (Svergun, 1999 ▸), *DAMMIF* (Franke & Svergun, 2009 ▸) and *GASBOR* (Svergun *et al.*, 2001 ▸). All these shapes are superimposed with the available crystal structures of these proteins taken from the appropriate Protein Data Bank (PDB) entries. To illustrate the performance of *SUPALM* on EM maps, a bead model generated directly from the experimental EMD map is superposed with the high-resolution cryoEM-derived model of the human gamma-secretase (Sun *et al.*, 2015 ▸). As an example of a heterogeneous assembly the model of the protein–RNA distribution in the 70S ribosome of *Escherichia coli* derived from X-ray and neutron scattering data (Svergun, Burkhardt *et al.*, 1997 ▸) is compared with the recent cryo-EM structure of the ribosome (Mitra *et al.*, 2006 ▸). As seen from Tables 1 ▸ ▸–3 ▸, for all these different objects the NSD values yielded by *SUPCOMB* agree well with the NSDs computed from the models provided by *SUPALM*. In Figs. 1 ▸ ▸–3 ▸ the template models and those superimposed by *SUPCOMB*/*SUPALM* are presented to illustrate the similarity of the results of the two programs. Note that, for the case of human gamma-secretase where the largest NSD values are observed, the atomic resolution fragments of the cryo-EM model were manually fitted inside the EMD map using *Coot* (Emsley & Cowtan, 2004 ▸) in the original publication (Sun *et al.*, 2015 ▸).

The results of Table 2 ▸ indicate that, in all test cases, a maximum number of spherical harmonics of *L*_max_ = 5 was sufficient to ensure a good-quality superposition of the two objects. If a lower number of harmonics are used, the matching quality becomes significantly worse (as can be judged from the higher NSD values), whereas higher *L*_max_ values increase the computation times but do not significantly improve the overlap.

The angular data range employed to compute the integrals in equation (5) can also be optimally selected. As illustrated in Table 3 ▸, *s_m_* values corresponding to seven Shannon channels *N*_sh_ (where *N*_sh_ = *D*_max_ *s_m_*/π and *D*_max_ is the maximum size of the particle) are sufficient for a reliable positioning. The use of wider angular intervals does not significantly improve the NSD values but, again, slows down the calculations. On the basis of these results the default values for *SUPALM* are selected to be *L* = 5 and *s_m_* = 7π/*D*_max_ (if needed, these values can be changed by the user from the command-line arguments).

## 5. Comparison of the performance of *SUPCOMB* and *SUPALM*   

*SUPALM* with the default parameters is about 1.5–2 times faster than *SUPCOMB* for small macromolecules with mol­ecular weights lower than 100 kDa (represented by about 10^3^ atoms). For large macromolecular complexes (*e.g.* 1 MDa, about 10^5^ atoms) *SUPALM* performs more than ten times faster compared to *SUPCOMB*. For superpositions of crystal structures with EM density maps the speed of *SUPALM* is of the same order of magnitude as that of the high-performance EM docking programs like *SITUS* or *ADP_EM*.

**Figure 4:**
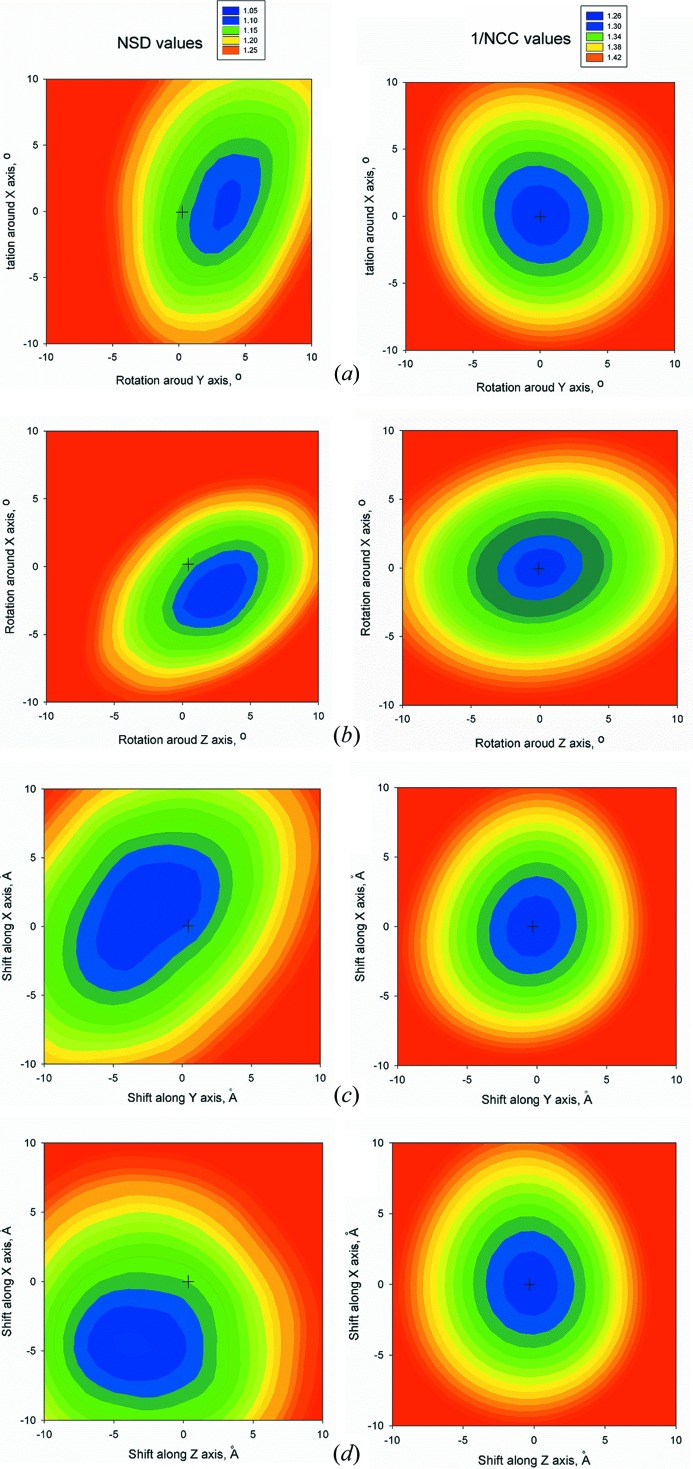
Two-dimensional contour plots of NSD (left column) and 1/NCC (right column) in the vicinity of the *SUPALM* solution for G protein (see Fig. 1 ▸ *b*). The *SUPALM* solution corresponds to the origin of the coordinates for all plots (marked with a cross symbol). (*a*), (*b*) The contour plots *versus* the rotations around *x*/*y* and *x*/*z* axes, respectively. (*c*), (*d*) The contour plots *versus* the shifts along *x*/*y* and *x*/*z* axes, respectively. The ‘true’ *SUPCOMB* solution (the minimum of the two-dimensional contour plot) is positioned close to the *SUPALM* solution (RMSD = 3.3 Å).

The superimposed models obtained by *SUPCOMB* and *SUPALM* are, as expected, not identical as the quantities to be minimized (NSD and 1/NCC, respectively) are different. Still, the overlaps in Figs. 1 ▸ ▸–3 ▸ and comparison of the NSD values in Table 1 ▸ indicate that, although the solutions by *SUPALM* reveal slightly higher NSD values, their positions essentially coincide with those given by *SUPCOMB*. Fig. 4 ▸ displays an example of NSD and 1/NCC contour profiles in the vicinity of a *SUPALM* solution. The plots of both functions display well defined minima, and both behave as analytical functions smoothly approaching the minimum values. Overall, in all the test examples, *SUPALM* provides practically the same results as *SUPCOMB* but runs much faster, especially for large macromolecular complexes.

We have also implemented an alternative procedure for speeding up the model superposition of *SUPCOMB*, using a different real-space metric for the proximity measure of three-dimensional objects, the normalized overlapped volume (NOV). Here, both objects are voxelized on a cubic grid in real space and the maximization of NOV provides the best matching of two objects. We found that the performance of the accelerated *SUPCOMB* using this metric is comparable to that of *SUPALM*; however, the NOV-based superpositions are not sufficiently sensitive to the finer details of the objects. For complicated shapes, the use of NOV may yield worse NSD values compared to *SUPALM* and to the standard (NSD-driven) mode of *SUPCOMB*. The rapid NOV mode may still be useful for high-throughput superpositions of simple shapes and will therefore be offered to *SUPCOMB* users.

An important feature of *SUPALM* (not available in *SUPCOMB*) is the ability to work directly with electron-density maps (EMD files) and with multi-phase *ab initio MONSA* models of inhomogeneous particles (*e.g.* protein–RNA and/or protein–DNA complexes). In the case of EMD models, the three-dimensional grid of voxels and the corresponding electron densities are directly used to calculate the scattering amplitudes; in the case of inhomogeneous bead models additional input files with the scattering density values for each bead phase (*e.g.* protein or DNA/RNA part of the complex) must be provided by the user. After the appropriate computation of the scattering amplitudes, the superposition runs automatically as in the plain bead or atomic model case.

## 6. Additional test cases for *SUPALM* alignments   

The examples considered in Table 1 ▸ utilized *ab initio* shapes restored from the experimental data to illustrate similarity between *SUPALM* and *SUPCOMB* results; therefore no ‘true’ positions of the structures were available, with *SUPCOMB* solutions used as reference. To test the ability of *SUPALM* to reconstruct ‘true’ alignments, an additional series of model calculations were performed. First, low-resolution bead envelopes were generated from the high-resolution structures in their reference positions. Then, the bead models were arbitrarily moved/rotated as rigid bodies and *SUPALM* was employed to reconstruct their initial position. In all cases presented in Table 1 ▸, the initial position was restored, with a r.m.s.d. of about 1 Å, demonstrating the ability of *SUPALM* to adequately find the best overlap. The small differences between the ‘true’ and matched structures are within the resolution limit used in the calculations.

**Figure 5:**
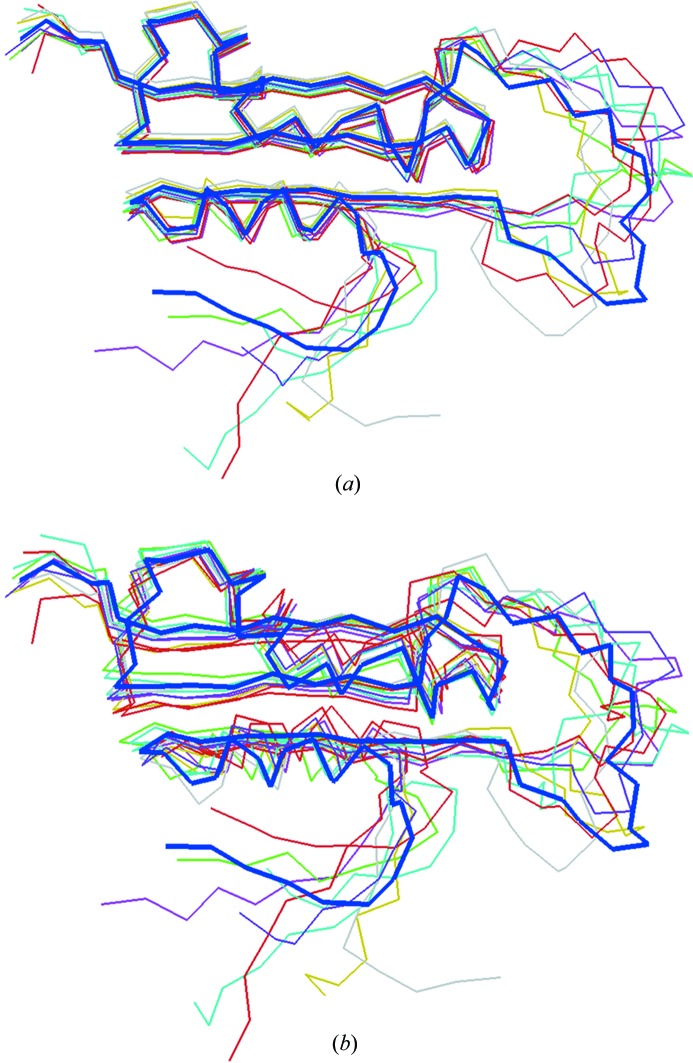
Different conformers of UVI31+ protein as determined by NMR (PDB code 2ma0) (*a*) and *SUPALM* alignments of these conformations onto the reference model (*b*). The reference NMR model is shown with a bold blue Cα trace in both panels.

Another test was conducted to check the stability of *SUPALM* when superposing variable conformations. For this, an NMR ensemble of protein structures (PDB code 2ma0 containing ten conformers of the small protein UVI31+; Chary, Rout & Rao, unpublished work) was selected. The first model in the PDB file was taken as a reference and all conformers were superimposed onto it by *SUPALM*. Fig. 5 ▸ displays the original NMR ensemble (where the rigid parts overlap with each other but the flexible portions display great variability) and the *SUPALM* superpositions, where the differences are, as expected, more uniformly distributed between the structures, but still the common parts of the ensemble structures are well superposed.

As a rule of thumb, for *SUPCOMB* alignments, NSD values around unity indicate good similarity between the two objects, whereas significantly larger values point to poor similarity. For NCC, the cut-off value for a good overlap depends on the molecular size, shape complexity and model resolution used in the calculations. From our test examples, the lower limit for a good similarity for NCC is around 0.7–0.8 at the resolution level used (*L* = 5 and seven Shannon channels).

## 7. Discussion and conclusions   

An algorithm for rapid automated superposition of high- and low-resolution models was proposed and tested on a number of macromolecular structures to demonstrate the reliability of the method. Contrary to *SUPCOMB* (Kozin & Svergun, 2001 ▸) where the CPU time is proportional to the product of the number of points in the two objects, the performance of the reciprocal-space superposition depends solely on the maximum number of spherical harmonics *L* and on the angular data range [0, *s_m_*] used to calculate the correlation coefficient. The default values of these parameters (*L* = 5 and seven Shannon channels in the range of integration) ensure a good quality of the superposition and at the same time provide a significant gain in the computing time against *SUPCOMB*. The gain is especially noticeable for large macromolecular structures where the Fourier space positioning becomes over ten times faster than in *SUPCOMB*.

The use of the normalized correlation coefficient based on the spherical harmonics representation employs a similar principle to the crystallographic fast rotation function method (Crowther, 1972 ▸), but contrary to the rotation function, we utilize only low-order harmonics at small angles to find the best overlap. Further, the number of harmonics in *SUPALM* can be defined by the user and it is not bound to a power of two as in the packages utilizing the fast Fourier transform technique (like *SITUS*, *FRM* or *ADP_EM*). We stress that the proposed method is not meant as an alternative to the mentioned EM packages, rather that the main aim of *SUPALM* is a rapid and convenient object matching at a level of the overall shape (*e.g.* using low-resolution models obtained from SAXS). Still, *SUPALM* permits the direct input and overlap of electron-density maps (EMD files) and multi-phase *ab initio* bead models (*MONSA*) and this significantly extends the applicability of the superposition method compared to *SUPCOMB*. *SUPALM* eliminates the need for intermediate steps (*e.g.* the transformation of the EMD file into a PDB model) and increases the accuracy of the superposition of heterogeneous structures (as the electron densities of each coordinate of the object are taken into account during the calculation of the scattering amplitudes, instead of using the simple approximation of a homogeneous particle). This option should facilitate a faster and more accurate comparison of SAXS models with those from complementary methods (such as EM, SANS, NMR and X-ray crystallography).

The proposed method reliably operates with default parameters, does not require user input and is therefore applicable in automated pipelines. The procedure, implemented in a program module *SUPALM* included in the *ATSAS* package (http://www.embl-hamburg.de/biosaxs/software.html), is freely available to academic users together with other programs from the *ATSAS* 2.7 release.

## Figures and Tables

**Figure 2:**
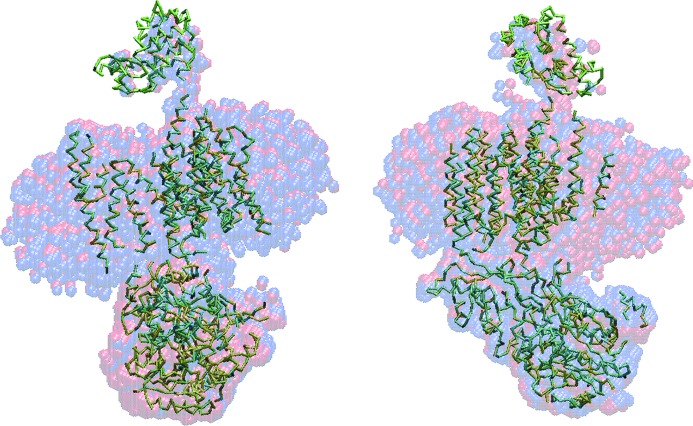
A hybrid high-resolution model of the human gamma-secretase derived from cryoEM (PDB code 4uis; Sun *et al.*, 2015 ▸) (green Cα traces) and the bead models from the electron-density map (ID EMD-2974.map) superimposed onto it by *SUPALM* (red spheres) and by *SUPCOMB* (blue spheres). The right view is rotated counterclockwise by 90° around the vertical axis.

**Figure 3:**
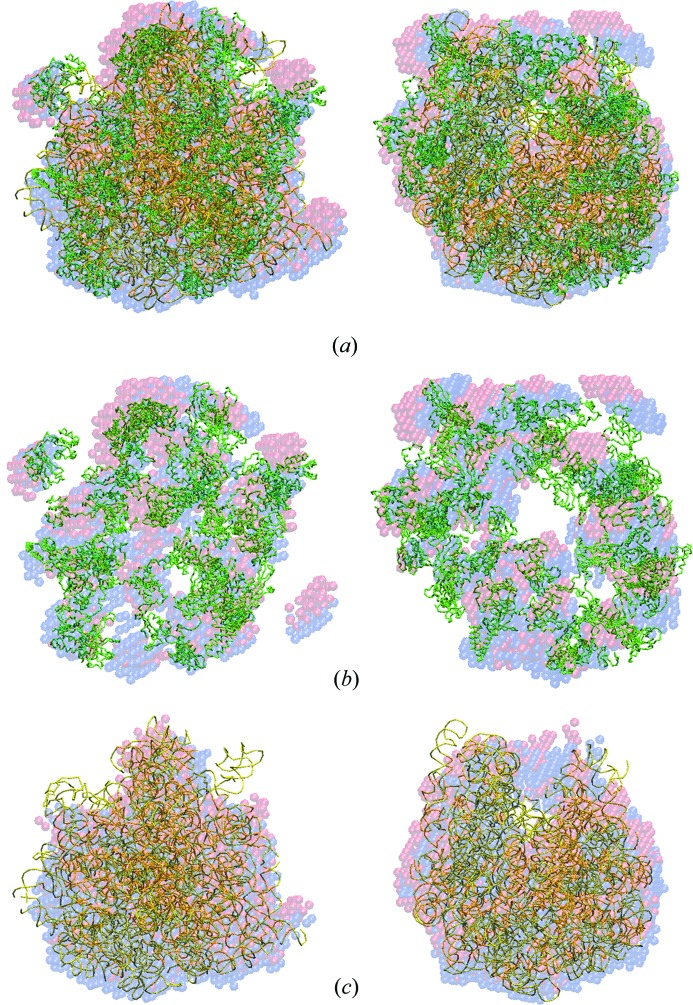
The crystal structure of the 70S ribosome (PDB code 4v4w; Mitra *et al.*, 2006 ▸) (green Cα traces correspond to the protein parts, the RNA parts are shown in yellow) with superimposed two-phase *ab initio* shapes obtained by *SUPALM* (red spheres) and by *SUPCOMB* (blue spheres). (*a*) The complete 70S ribosome models, (*b*) the protein parts, (*c*) the RNA parts. The right view is rotated counterclockwise by 90° around the vertical axis.

**Table 1:** Test objects superimposed using *SUPALM* and *SUPCOMB* The template objects represent high-resolution structures (their scattering amplitudes were calculated with *CRYSOL*) and the matched objects are low-resolution *ab initio* shapes (with the scattering amplitudes calculated by *DAM2ALM*) obtained by *DAMMIN* (Svergun, 1999 ▸), *DAMMIF* (Franke & Svergun, 2009 ▸), *GASBOR* (Svergun *et al.*, 2001 ▸) and *MONSA* (Svergun & Nierhaus, 2000 ▸). The NSD values were computed between the template and the matched objects obtained by *SUPCOMB* and *SUPALM*. The NCC values were evaluated by *SUPALM*. The computing gain factor is obtained as the ratio of CPU time of *SUPCOMB* to that of *SUPALM*. The default parameters of *SUPALM* (*L*_max_ = 5 and *N*_sh_ = 7) were used for all tests.

No.	Template object	Matched object	MW (kDa)	NSD, *SUPCOMB*	NCC, *SUPCOMB*	NSD, *SUPALM*	NCC, *SUPALM*	CPU gain factor *SUPALM* *versus* *SUPCOMB*
1	2a38 (PDB)	z1z2 (*DAMMIF*)	21	1.70	0.87	1.79	0.96	1.6
2	1got (PDB)	G protein (*GASBOR*)	40	1.12	0.72	1.14	0.76	2.8
3	1o6v (PDB)	Internalin (*DAMMIN*)	50	1.69	0.74	1.94	0.95	3.2
4	4uis (PDB)	EMD-2974 (EMD map)	140	3.65	0.86	3.75	0.93	70.0†
5	1hqk (PDB)	Lumazine synthase (*DAMMIN*)	960	1.22	0.83	1.24	0.87	10.1
6	4v4w (PDB)	70S ribosome (*MONSA*)	2150	1.10	0.81	1.27	0.94	25.2

† For the EMD map file (EMD-2974) the comparison with *SUPCOMB* was made in the following way. For *SUPCOMB* the electron-density map was first transformed into a bead PDB file (containing 29 374 atoms) using the density threshold 0.04 by the program *EM2DAM* from the *ATSAS* package and then it was superimposed with the PDB structure (4uis). *SUPALM* performed the direct superposition of PDB and EMD files. The RMSD between the deposited fitted EMDataBank model and the *SUPALM* fit is 5.5 Å.

**Table 2:** Test objects superimposed by *SUPALM* with different maximum number of harmonics *L*_max_ used in the calculations of the scattering amplitudes The objects are the same as in Table 1 ▸. The angular data range corresponding to seven Shannon channels (*N*_sh_ = 7) was used for the tests. Values in bold correspond to the default setting for the maximum number of harmonics *L*_max_.

No.	Template object	Matched object	MW (kDa)	NSD/NCC (*L*_max_ = 3)	NSD/NCC (*L*_max_ = 4)	NSD/NCC **(*L*_max_ = 5)**	NSD/NCC (*L*_max_ = 6)	NSD/NCC (*L*_max_ = 7)
1	2a38 (PDB)	z1z2 (*DAMMIF*)	21	1.82/0.97	1.84/0.96	**1.79/0.96**	1.80/0.95	1.79/0.95
2	1got (PDB)	G protein (*GASBOR*)	40	1.28/0.77	1.16/0.76	**1.14/0.76**	1.14/0.75	1.13/0.74
3	1o6v (PDB)	Internalin (*DAMMIN*)	50	2.08/0.97	1.96/0.96	**1.94/0.95**	1.95/0.94	1.93/0.93
4	4uis (PDB)	EMD-2974 (EMD map)	140	4.10/0.95	3.89/0.94	**3.75/0.93**	3.75/0.92	3.74/0.91
5	1hqk (PDB)	Lumazine synthase (*DAMMIN*)	960	1.27/0.88	1.26/0.87	**1.24/0.87**	1.25/0.87	1.24/0.86
6	4v4w (PDB)	70S ribosome (*MONSA*)	2150	1.30/0.96	1.28/0.95	**1.27/0.94**	1.28/0.94	1.26/0.93

**Table 3:** Test objects superimposed by *SUPALM* using different angular data ranges *N*_sh_ for the calculations of the scattering amplitudes The objects are the same as in Table 1 ▸. The default number of spherical harmonics (*L*_max_ = 5) was used for the tests. Values in bold correspond to the default setting for the angular data range.

No.	Template object	Matched object	MW (kDa)	NSD/NCC (*N*_sh_ = 5)	NSD/NCC (*N*_sh_ = 6)	NSD/NCC (*N*_sh_ = 7)	NSD/NCC (*N*_sh_ = 8)	NSD/NCC (*N*_sh_ = 9)
1	2a38 (PDB)	z1z2 (*DAMMIF*)	21	1.95/0.97	1.87/0.96	**1.79/0.96**	1.80/0.95	1.79/0.94
2	1got (PDB)	G protein (*GASBOR*)	40	1.17/0.77	1.16/0.76	**1.14/0.76**	1.15/0.76	1.14/0.75
3	1o6v (PDB)	Internalin (*DAMMIN*)	50	2.13/0.96	2.02/0.95	**1.94/0.95**	1.96/0.94	1.95/0.94
4	4uis (PDB)	EMD-2974 (EMD map)	140	5.33/0.95	3.91/0.94	**3.75/0.93**	3.74/0.93	3.73/0.92
5	1hqk (PDB)	Lumazine synthase (DAMMIN)	960	1.28/0.88	1.25/0.87	**1.24/0.87**	1.24/0.87	1.23/0.86
6	4v4w (PDB)	70S ribosome (*MONSA*)	2150	1.30/0.97	1.29/0.95	**1.27/0.94**	1.28/0.94	1.27/0.93
